# 

**Published:** 1917-05-12

**Authors:** 


### CONTENTS.

| | PAGE |
|---|---|
| **Mens Sana in Corpore Sano** ... ... ... | 101 |
| **The Medical Profession and Legislation Against Contraceptives** ... ... ... ... | 1C2 |
| **Hospital and Institutional News** ... ... ... | 103 |
| Medical Officers for the R.A.M.C.—Medical Protection—The Late Dr. J. E. Squire—General Dismay at the Shortage of Doctors—India's Red Cross Gift—A Courageous Appeal—Large Sums for Midland Charities—A Doctor's Important Bequest—"The Silvertown Cot"—The Prince of Wales' Hospital for Limbless Sailors and Soldiers, Cardiff—Staff Puzzles in Wartime—One Thousand Sanatorium Beds in Wales—Hospital Finance—St. Andrew's Hospital, Dollis Hill—Life Assurance of Medical Men on War Service—An Unsolved Problem of Public Health—The Unhealthy Cinema—The Manufacture of Artificial Limbs—Epidemics in Roumania—The Puerperal Death-rate in Anglesey—Average Occupied Beds: One—Non-Resident In-Patients—Hospital Staffs and the Tribunals—The Death of a Veteran Guardian—A Deputation to Lord Rhondda—A School Clinic at Leeds—War Work and Women's Health—A Large Estate Going Begging — Resignation of the Matron of Worcester General Infirmary—Hospital Extension at Ashton-under-Lyne—Quinine for the Army—This Week's Drug Market. | |
| **Child Mortality and Child Welfare** ... ... | 109 |
| By Everitt E. Norton, M.D., D.P.H. | |
| **Science and Pseudo-Science** ... ... ... | 110 |
| The Hereditary Criminal Again? | |
| **National Insurance Act Developments...** ... | 111 |
| National Insurance and the Proposed Ministry of Health. | |
| **The Doctor's Vade-Mecum** ... ... ... | 113 |
| Simple Rules for Medical Practitioners. | |
| **Hospital Pharmacists and Food Economy** ... | 114 |
| **Hospital and Institutional Results** ... ... | 114 |
| **The Matrons' and Sisters' Department** ... | 115 |
| Plain Words on Registration.—V. | |
| Round the Hospitals. | |
| Miss Luckes, R.R.C.—Miss Amy Hughes' Resignation—The Nation's Fund for Nurses—Embryo Probationers' Perils—How to Save Them—A Public Warning Needed. | |
| **Food in Wartime** ... ... ... ... ... | 118 |
| M.A.B. Economy: Its Excellent Results. | |
| **The Patriotism of the Nurse-Training Schools** | 119 |
| XIV.—Some Yorkshire Institutions. | |
| **Matrons' and Sisters' Appointments** ... ... | 120 |
| **Presentation** ... ... ... ... ... ... | 120 |
| **Parliament and the Profession** ... ... ... | 120 |
| **Gifts and Bequests** ... ... ... ... ... ... | 120 |
| **Annual Meetings** ... ... ... ... ... ... | 120 |
| **The Institutional Worker** ... ... (Suppl.) | 1 |
| Prevention of Waste in Foodstuffs. | |
| How to Prevent Infection in a Surgical Ward. | |

